# Profiled Ion Exchange Membranes: A Comprehensible Review

**DOI:** 10.3390/ijms20010165

**Published:** 2019-01-04

**Authors:** Sylwin Pawlowski, João G. Crespo, Svetlozar Velizarov

**Affiliations:** Associated Laboratory for Green Chemistry - Clean Technologies and Processes (LAQV), REQUIMTE, Chemistry Department, FCT, Universidade NOVA de Lisboa, 2829-516 Caparica, Portugal; s.pawlowski@fct.unl.pt (S.P.); jgc@fct.unl.pt (J.G.C.)

**Keywords:** ion exchange membranes, profiled membranes, corrugated membranes, electrodialysis, reverse electrodialysis, membrane capacitive deionization, hydrodynamic, mass transfer, thermal pressing, 3D printing

## Abstract

Profiled membranes (also known as corrugated membranes, micro-structured membranes, patterned membranes, membranes with designed topography or notched membranes) are gaining increasing academic and industrial attention and recognition as a viable alternative to flat membranes. So far, profiled ion exchange membranes have shown to significantly improve the performance of reverse electrodialysis (RED), and particularly, electrodialysis (ED) by eliminating the spacer shadow effect and by inducing hydrodynamic changes, leading to ion transport rate enhancement. The beneficial effects of profiled ion exchange membranes are strongly dependent on the shape of their profiles (corrugations/patterns) as well as on the flow rate and salts’ concentration in the feed streams. The enormous degree of freedom to create new profile geometries offers an exciting opportunity to improve even more their performance. Additionally, the advent of new manufacturing methods in the membrane field, such as 3D printing, is anticipated to allow a faster and an easier way to create profiled membranes with different and complex geometries.

## 1. Introduction

Electro-membrane processes range from classical membrane electrolysis and electrodialysis to emerging applications, such as reverse electrodialysis, membrane capacitive deionization, redox flow batteries, microbial, and enzymatic fuel cells, and ion exchange membrane (bio)reactors. There is an increasing worldwide interest in their use for clean water and renewable electrochemical energy harvesting and storage [1,2,3,4,5,6,7], which has inspired fundamental and applied research on improving the properties of their “key” components: the ion exchange membranes (IEMs).

Academia and industrial companies are usually focused on the development of tailored membrane products best suited to satisfy specific process requirements, such as low membrane electric resistance, monovalent ion perm-selectivity and/or antifouling properties [8,9,10]. Thus, material properties of IEMs have been mainly studied, while the design and hydrodynamic of electromembrane devices have suffered few alterations since the traditional plate-and-frame arrangement of flat membranes and spacers have been, so far, the most common choice [11,12].

Recently, profiled membranes (also known as corrugated membranes, micro-structured membranes, patterned membranes, membranes with designed topography or notched membranes) are gaining more attention, mostly due to advances in their manufacturing, as well as improvement of their performance, which depend on the shape, dimensions, orientation, and distribution of the microscopic profiles (corrugations) on their surfaces. According to our best knowledge, based on the information provided by Zabolotskii et al. [13], the idea of creating profiled ion-exchange membranes (i.e., with both membrane and spacer functionalities) emerged and was firstly implemented in the decades of 70–80 of the 20th century in the former Union of Soviet Socialist Republics (U.S.S.R) with the objective (successfully achieved) of accelerating the mass transfer processes in a electrodialyzer, by increasing the available membrane surface area. Later, in 2009, Brauns [14] suggested to use profiled ion exchange membranes also in reverse electrodialysis stacks in order to decrease their height, and consequently, the pressure drop. Veerman et al. [15] proposed a fractal profiled membranes design for reverse electrodialysis (RED) application to reduce the flow path in order to maximize salinity gradient.

In this review, we will focus especially on the developments and applications of profiled ion exchange membranes during the last 10 years, which is also the most productive period regarding their investigation. A critical discussion about the advantages and disadvantages of their use will be provided in the context of their application in electrodialysis (ED) and reverse electrodialysis (RED). We are also propose and discuss the possibility of using profiled membranes in membrane capacitive deionization (MCDI), which could open opportunities for improving this novel process. Additionally, a summary of the possible patches of preparing profiled membranes is presented, with a special focus on additive manufacturing (3D printing) and its possible symbiosis with computational fluid dynamics (CFD) studies.

## 2. Profiled Ion-Exchange Membranes (10 Years Ago)

In 2008, Larchet et al. [16] compared the performance of electrodialysis and electrodeionization stacks with non-conductive spacers, profiled ion exchange membranes and ion-exchange resins. Utilization of profiled membranes allowed for an effective production of drinking water from brackish waters with larger salt concentration ranges than that achieved by other stack arrangements. Utilization of profiled membranes obviates the need of using non-conductive spacers, thus increasing the membrane surface area available for mass transfer. Also, a curved shape of profiled membrane surface could facilitate initiation of electroconvection, which is favoured in dilute saline solutions and allows for a better fluid mixing near the profiled membrane surface. Utilization of profiled membranes for desalination of solutions with low conductivity showed to be more advantageous than the use of non-conductive spacers, due to the reduction of electric resistance in the compartment. Grabowski [17] reported final reduction of Reverse Osmosis (RO) permeate conductivity to approximately 2 µS/cm with profiled membranes, in comparison to approximately 5 µS/cm when flat membranes and spacers were used. Moreover, although there might exists a bipolar junction in the contact points between profiled anion and cation exchange membranes where dissociation of water could be catalysed, it has been pointed out that since the maximum local current density becomes lower when profiled membranes are used, due to a higher available membrane area, the water splitting rate decreases, which is very promising for production of high purity water [18]. Overall, the rate of mass transfer increased significantly (by a factor of four) when profiled membranes were used, while the pressure drop was similar to the one when non-profiled membranes were used.

**Figure 1:**
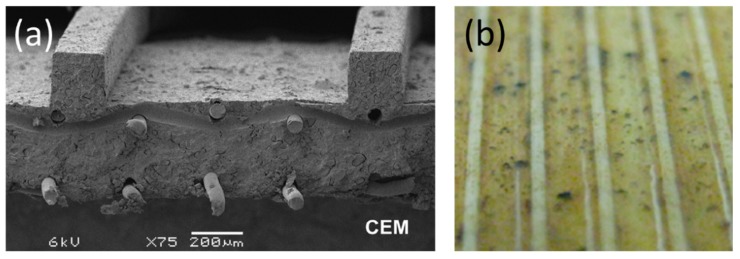
Profiled membranes with straight ridges parallel to the flow direction: (**a**) SEM-image of a cross-section of the profiled cation-exchange membrane (CEM). Reproduced with permission from Reference [19]. Copyright 2011 Elsevier; (**b**) Photo of 1 × 1 cm^2^ section of profiled CEM after more than 60 days of reverse electrodialysis (RED) stack operation, in which air sparging was applied as an antifouling strategy. Reproduced with permission from Reference [21]. Copyright 2016 American Chemical Society.

In 2011, Vermaas et al. [19] first performed experimental tests in RED with profiled membranes (reliefs were straight ridges oriented parallelly to the flow direction, see Figure 1a). A 10% higher net power density was obtained when profiled membranes were used in comparison to the stack with non-conductive spacers. The main improvements of using those profiled membranes were the decrease by 30% of total electric resistance (mainly due to elimination of spacer shadow effect) and a four times lower pressure drop. However, the diffusion boundary layer resistance (at a Reynolds number of 5, when the highest net power density was obtained) was around 4.25 times higher when profiled membranes were used (in comparison to the stack with spacers), since the solution mixing promoted by profiled membranes was less efficient than the one offered by the spacers. Despite that, the stacks with this particular profiled membrane were significantly less sensitive to fouling than stacks with spacers [20], and easy to be cleaned [21] (Figure 1b), since the straight and parallel to the flow relief of profiled membranes created open channels for fluid flow, where the foulants struggled to get deposited.

The comparison between profiled membranes and spacers, as well as the advantages and disadvantages of each one, obviously strongly depend on the shape of the reliefs, as well as the quality of the spacers mesh. Moreover, the specific process operating conditions, a relatively high current density, and higher flow rates used in ED, and a low current density and lower flow rates applied in RED, influence the differences in hydrodynamic and mass transfer existing when spacers or profiled membranes are used. Thus, for each application, and conditions, a different reliefs shape might prove to be the optimal one, since the objectives of different electromembrane processes are distinct. In ED, the main objective is to intensify the mass transfer, while in RED, it is desired to obtain high net power density values, i.e., to maintain a low pressure drop (requiring less energy demand for pumping) at a reasonably mass transfer rate.

## 3. Progresses in the Performance of Profiled Ion-Exchange Membranes

**Figure 2:**
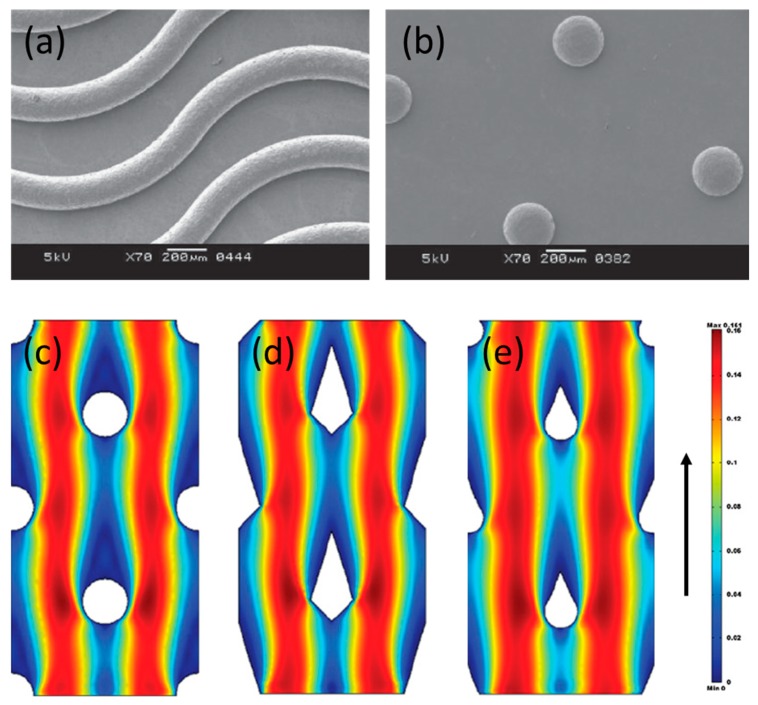
Surface morphology of tailor-made profiled membranes with: (**a**) waves and (**b**) pillars corrugations. Reproduced with permission from Reference [22]. Copyright 2014 Elsevier.; velocity fields, obtained by computational fluid dynamics (CFD), around pillar structures with (**c**) round, (**d**) diamond, and (**e**) tear shape, for a flow direction indicated by the upward arrow on the right. Reproduced with permission from Reference [25]. Copyright 2010 Elsevier.

Guler et al. [22] investigated experimentally the performance of profiled membranes with corrugations similar to straight-ridges, waves, and round pillars in RED stacks (Figure 2a,b). A 20% higher net power density (in comparison to a stack with spacers) was obtained when pillar profiled membranes were used, which also outperformed the other studied profiles, mainly due to the lowest pressure drop observed in channels with pillar profiled membranes, and the lower increase of the diffusion boundary layer resistance (it was still 1.5 times higher but, when straight-ridges were used at the same operating conditions, it was 2.25 higher). Similar beneficial effects of hemispherical protrusions, similar to pillar profiled membranes, were observed by Liu et al. [23] when used in a microbial RED stack. Gurreri et al. [24] simulated, by CFD tools, the performance of profiled membranes with squared and round pillars. Round pillars were anticipated to perform better than squared pillars and their simulations are in accordance with previous findings, since the pumping power consumption is expected to be lower when profiled membranes are used in comparison to the use of spacers. However, they also observed the development of stagnant zones near the pillar corrugations, which can be responsible for concentration polarization (and consequently, a higher diffusion boundary layer resistance). Moreover, it was observed that pillar-kind corrugations, independently of their exact shape (round, diamond, tear, etc.), lead to development of zones with poor mixing upstream of the corrugations, which are very prone for deposition of foulants [25] (Figure 2c–e).

**Figure 3:**
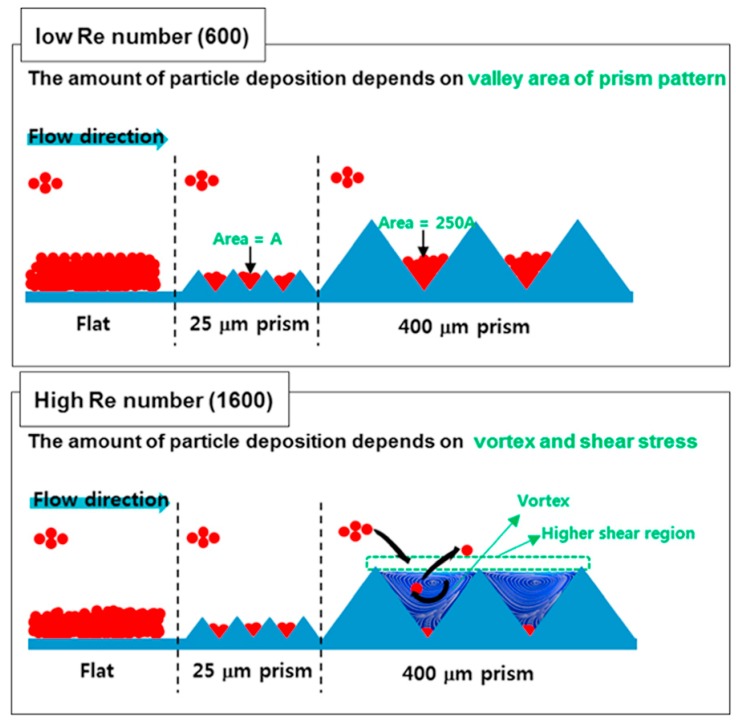
An illustrative scheme of correlation of membrane fouling with topography of profiled membranes in cross-flow filtration for water treatment. Re stands for Reynolds number. Particles are represented in red and the prismatic membrane surface pattern by blue triangles. The bending arrows illustrate particles entering and leaving locally created flow vortexes Reproduced with permission from Reference [33]. Copyright 2016 Elsevier.

Fouling is a major issue in any membrane-based process, including the electromembrane ones [26,27,28,29]. However, there are almost no reported experimental studies performed with natural waters using profiled ion-exchange membranes, except for the already previously mentioned works of Vermaas et al. [20,21]. In pressure-driven membrane-based processes, it has been seen that surface patterning by nanoimprints made from the same material as membranes [30,31], or an anti- or less-prone-to-fouling material such as titanium dioxide (TiO_2_) [32], has fouling mitigation effects. Also, based on computational and experimental studies, some trends about the possible relation between fouling and flow behaviour in channels formed by profiled membranes were obtained. For example, utilization of prism corrugations equidistant by 400 µm in cross-flow filtration enhanced vortex formation and reduced the mass of deposited foulants by around seven times in comparison to flat membranes at Re = 1600 [33]. Increasing the wall shear stress was another effective strategy for minimization of foulants deposition, which can be achieved by optimizing the shape of corrugations [34] and/or increasing the linear flow velocity (Figure 3).

Zhao et al. [35] performed CFD simulations of notched membranes with different distribution of squared corrugations on the membrane surface used in ED and observed that better hydrodynamic conditions result from an increase of the flow velocity perpendicular to the membrane, which leads to reduction of concentration polarization. However, they also observed that if the corrugations are very close to each other, the flow behaviour becomes very similar to that of flat membranes, thus, the best distance between corrugations and the size of their side was found to be equal to 120 µm.

**Figure 4:**
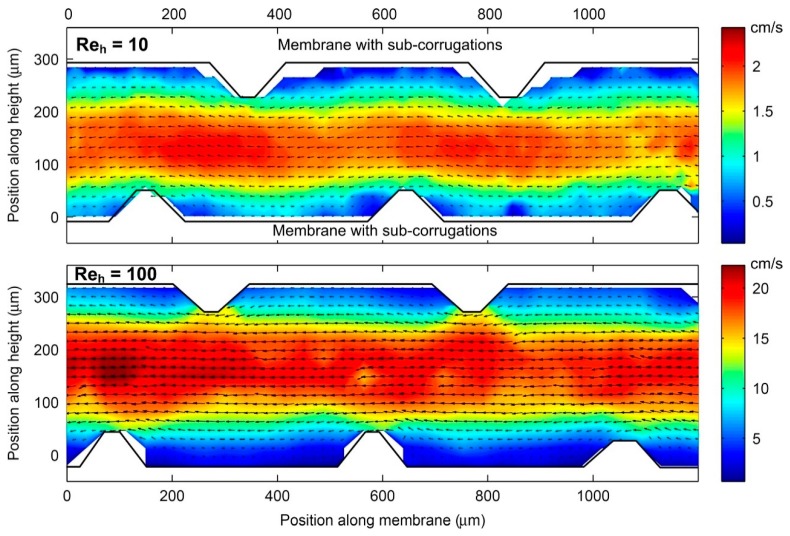
Velocity fields, obtained by particle tracking velocimetry (PTV), of fluid flow between sub-corrugated membranes for Reynold numbers (based on half the channel height), Re_h_ of 10 and 100 in a RED application. Reproduced with permission from Reference [36]. Copyright 2014 Elsevier.

Vermaas et al. [36] included sub-corrugations perpendicular to the flow in the channels formed by straight-ridge profiles in a RED stack. However, such sub-corrugations did not create any vortices since the velocity range in RED is too low. Instead, dead zones were formed in front and behind the sub-corrugations, as observed by particle tracking velocimetry (PTV) (Figure 4). However, in ED, higher flow rates and current densities (close to limiting and/or over-limiting current density) are used [37], thus utilization of such sub-corrugations might promote electro-osmotic chaotic fluid instabilities, and consequently enhance mass transfer [38,39,40]. Imprinting microgel patterns on membranes surfaces [41], screening them with nonconductive strips [42,43] or coating by hydrophobic materials [44] can also promote formation of such electro-convective vortices.

**Figure 5:**
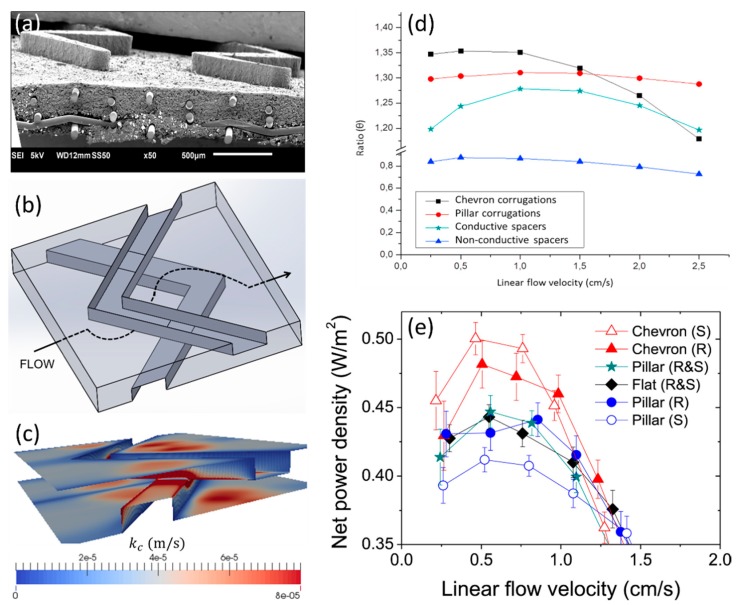
Chevron profiled membranes used in RED: (**a**) SEM image of the surface morphology, (**b**) segment of a channel formed by chevron profiled membranes; (**c**) map of mass transfer coefficient, obtained by CFD, at two linear flow velocity of 1.0 cm/s, (**d**) comparison, based on CFD simulations, of the ratio (θ) between net power density obtainable in RED stacks with different configurations and the one obtainable in RED stack with empty channels, (**e**) experimental net power density obtained in RED stacks with different configurations. Reproduced with permission from References [45,46]. Copyright 2016 and 2017, respectively, Elsevier.

Pawlowski et al. [45], based on CFD studies, proposed the use of profiled membranes with chevron (V-shaped) profiles for RED (Figure 5a). Instead of focusing on decreasing pressure drop, the approach followed was mainly focused on improving the mass transfer. To create a channel for fluid flow, two profiled membranes (bottom and top) should be aligned and stacked with the chevron tips pointed into the opposite directions. In such a way, a very specific fluid pathway is created (the fluid goes periodically up and down, and it is also distributed in the lateral directions), which promotes convective phenomena (Figure 5b). Additionally, the linear flow velocity upstream to the corrugations is high, which intensifies the mass transfer and should avoid accumulation of foulants in such critical areas (Figure 5c). Although the pressure drop in channels with chevron profiled membranes was anticipated to be higher (in comparison with pillar profiled membranes), the net power density values were anticipated to be higher (for linear flow velocities lower than 1.5 cm/s) (Figure 5d), since chevron corrugations promotes fluid mixing more effectively near the membrane interface, which enhances local mass transfer. Besides the profiles shape, it was predicted that the maximum net power density values also depend on the stack length. For a 10-cm long stack, chevron profiled membranes and linear flow velocity between 0.5 and 1.0 cm/s were expected to be the best options. These predictions were confirmed experimentally and an 8–14% increase of net power density was observed when chevron profiled membranes were used instead of pillar profiled membranes or non-conductive spacers [46] (Figure 5e). The pillar profiled membranes and non-conductive spacers had a similar performance, since state-of-the art spacers (with very fine, elastic, and flexible filaments) were used in that study.

Additionally, Pawlowski et al. [46] proposed a further simplification of chevron corrugations into “chevron-crossed” corrugations, which is expected to facilitate assembling of large stacks, although at “ideal” conditions, simulated by CFD, they would promote less convective phenomena than the original chevron profiled membranes (but still more than pillar profiled membranes). La Cerva et al. [47] simulated by CFD tools the performance of profiled membranes similar to the “chevron-crossed” corrugations (the corrugations instead of rectangular are semi-cylindric), and referred to them as “Overlapped Crossed Filaments” (OCF). The expected Sherwood number for chevron-crossed and OCF corrugations, at flow angle attack of 45°, and at the lowest simulated Re number is 10.7 [46] and 12.6 [47], respectively, thus semi-cylindrical corrugations are expected to offer a slightly better performance. Further optimizations for RED applications must also have in attention that the same profiled membranes might not always have the same beneficial effects, as, e.g., the dilute feed stream concentration influences the global stack performance [48]. For example, when compared with empty channels, inclusion of profiled membranes leads to higher net power density values if the dilute saline stream concentration is 0.01 M or lower, as this decreases significantly the electric resistance of the dilute saline solution compartment.

**Figure 6:**
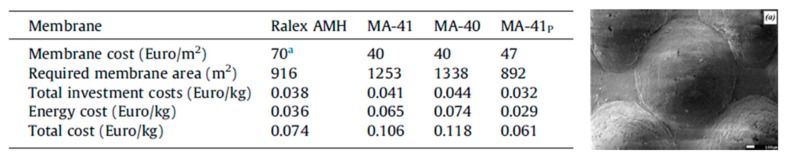
Economic evaluation of an ammonium nitrate treatment process using different anion-exchange membranes (AEM), in which Ralex AMH, MA-41 and MA-40 are flat, MA-41p is a profiled (subscript “p”) version of MA-41, which surface denoted by *(a)* is shown on the right with a scale bar of 100 μm. ^a^Prices are given for the year 2011. (Reproduced with permission from Reference [49]. Copyright 2016 Elsevier).

Melnikov et al. [49] tested profiled membranes, which surface appears to be all covered by corrugations with a shape of hemispheres (in the previously discussed cases there are clear gaps and the corrugations are spaced between each other), in an ED treatment of secondary steam condensate obtained during production of ammonium nitrate. Again, the profiled membranes allowed for an enhanced mass transfer, in comparison to that of using flat membranes made from the same material, due to the reasons already discussed in Section 2. Moreover, the total cost of ammonium nitrate treatment decreased from 0.106 €/kg to 0.061 €/kg when profiled membranes were used (in comparison with flat membranes), since the required membrane area decreased by 30% and the estimated additional cost of preparing such profiled membranes by thermal pressing is low (just 7 €/m^2^) (Figure 6). Vasil’eva et al. [50] tested such profiled membranes in a diffusion dialysis process for separation and purification of amino acids. A maximum 8-fold increase of phenylalanine flux was documented, when profiled instead of flat membranes were used, since the available membrane area for mass transfer increased 2.3 times and the diffusion boundary layer thickness was reduced due to more favourable hydrodynamic conditions, resulting from the curvature of the membrane surface.

## 4. Profiled Membranes for Membrane Capacitive Deionization (MCDI)

As it can be seen, utilization of profiled membranes in ion-exchange membrane processes such as electrodialysis, electrodeionization, reverse electrodialysis, and diffusion dialysis have led to improvements in their performance in comparison to the utilization of flat membranes. We are therefore suggesting that the next electromembrane process, in which profiled ion-exchange membranes might have a bright future could be membrane capacitive deionization (MCDI). Membrane capacitive deionization cell architecture consists in modifying the classical capacitive deionization (CDI) cell design by placing an appropriate ion-exchange membrane in front of each electrode (CEM near cathode and AEM near anode). In such arrangement, the respective membranes block co-ions transport (i.e., occurrence of parasitic current), thus leading to improvement of charge efficiency and an increase of salt storage in the macropores of the corresponding electrodes [51]. However, the inclusion of membranes adds also an additional electric resistance (of the membranes themselves and of the associated diffusion boundary layers) to the system. Therefore, utilization of profiled ion-exchange membranes, besides granting a selective counter-ion transport, might also offer a larger area for mass transfer and a lower diffusion boundary layer resistance. Moreover, such beneficial effects are enhanced (as seen for the case of electrodialysis) at high feed streams flow rates, low solutions concentrations and high current densities, which are also the operating conditions relevant to MCDI.

## 5. Preparation of Profiled Ion Exchange Membranes

**Figure 7:**
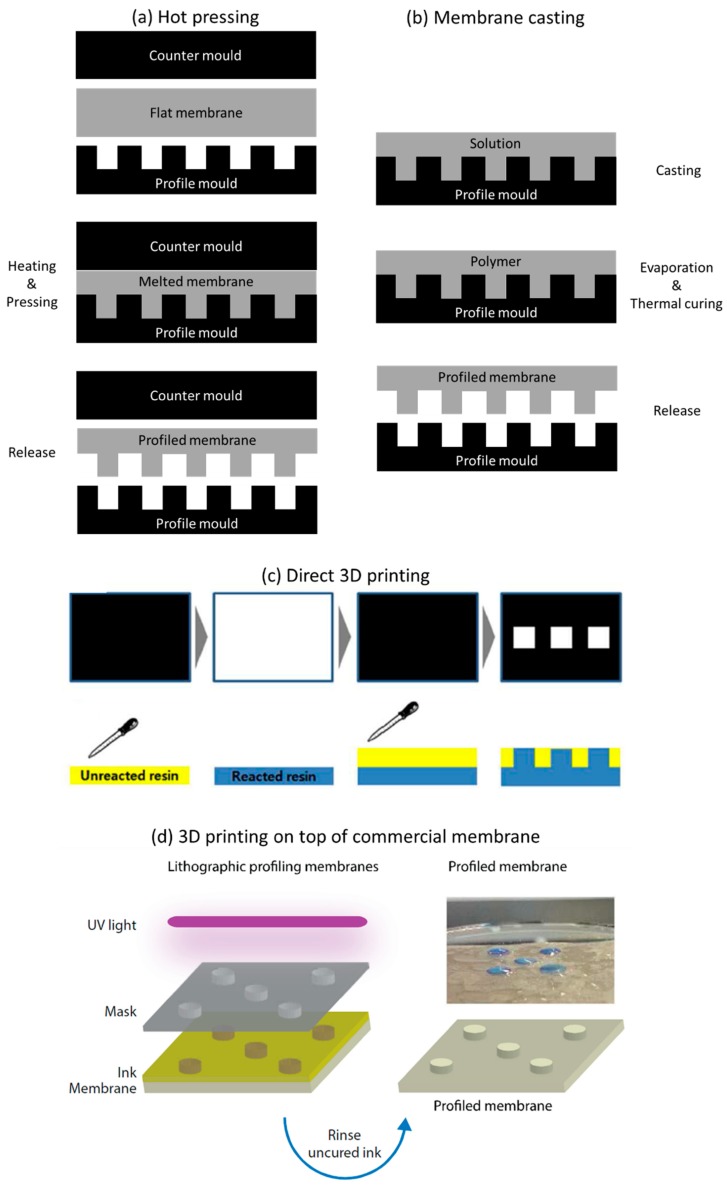
Different possible pathways for preparation of profiled ion exchange membranes: (**a**) hot pressing; (**b**) membrane casting (Adapted from Reference [22]); (**c**) Direct 3D printing (Reproduced with permission from Reference [52]. Copyright 2016 American Chemical Society.); (**d**) 3D printing on top of a commercial membrane (Reproduced with permission from Reference [53]. Copyright 2018 Timon Rijnaarts).

Profiled ion exchange membranes can be prepared by three main approaches: hot (thermal) pressing (Figure 7a), membrane casting (Figure 7b), and 3D printing (photopolymerization) (Figure 7c,d).

Hot pressing (Figure 7a) is a relatively easily preparation method, in which a dry flat heterogenous ion exchange membrane is first sandwiched between moulds with a desired subsequent corrugations shape and then placed in a thermal press, where the whole set-up is heated. While the membrane material melts, the moulds are pressed against each other, thus originating formation of the corrugations while the area between them is compressed [19,46]. In this way, when cooled, the new shape of the profiled membrane is formed. Releasing a profiled membrane from the mould is a delicate procedure, but it can be facilitated by using releasing agents, which are initially sprayed on the flat membrane surface [19]. Also, in order to assure an easier and complete release of the membranes from the moulds, without breaking the corrugations, their height should not be bigger than their width [46]. Alternatively, instead of using moulds, spacers can be pressed against the membranes to create channels on their surface [35]; however, the resolution of the corrugations shape and their dimensions are lower.

Since membranes dry when heated and pressed, if they are then brought into contact with an aqueous medium, they will swell. The corrugations overall shape remains the same, but their dimensions increase (differently for AEM and CEM, according to their materials swelling tendency) [46]. The concentration of saline streams also influences membranes swelling (higher volumetric changes are observed for membranes in contact with dilute solutions) [54]. Therefore, this phenomenon must be considered when the moulds’ dimensions are designed. Recently, hot pressing of previously swollen flat membranes was tested but, unfortunately, the data regarding corrugations fidelity are not available [49]. Nevertheless, since the fraction of the surface area occupied by ion exchange resin particle was 25% for a profiled membrane swollen a priori, and just 12% for a profiled membrane obtained from a dry material (for a flat membrane the mentioned fraction was 15%), the mass transfer surface was twice higher [50].

Hot pressing of the membranes might lead to some alteration of their material properties [13,55], but usually they are beneficial. For example, the conductivity and effective specific resistance of Ralex AMH-PES heterogeneous anion-exchange membrane increased from 9.1 to 13.9 mS/cm [19] and decreased from 195 to 111 Ωcm [46], respectively, but no significant alterations were observed after hot pressing of a Ralex CMH-PES heterogeneous cation-exchange membrane. Most probably, during melting, the percolating pathway through the Ralex AMH-PES membrane was improved. Only heterogeneous ion exchange membranes could be profiled by hot pressing since homogeneous ion exchange membranes are often cross-linked, thus cannot be melted.

Membrane casting (Figure 7b) offers the possibility of preparing profiled membranes from a homogeneous material. The materials and operating conditions used for preparation of a profiled homogeneous ion exchange membrane are the same as for a flat membrane with the following difference: the initial membrane solution is casted onto a corrugated mould (could be the same as the one used for hot pressing) instead on the surface of a flat plate [22,23]. The thickness of the non-profiled parts of the membrane (therefore the membrane base) can be adjusted by changing the volume or concentration of the membrane-forming solution spread on the mould, while the size of the corrugations depends from the mould shape. The so far used membrane casting of profiled ion exchange membranes was always based on phase inversion (meaning solvent evaporation and solidification of membrane material in the mould) [22,23]. The disadvantage of membrane casting over hot pressing is the difficulty of releasing the profiled membranes formed from the mould, as many of them break during the process. Alternatively, a spacer structure can be replicated on a membrane by “capillary force induced surface structuring” [56], in which the top layer (of an adjustable thickness) is modified by placing a spacer on its surface, while the solvent is evaporated (therefore the membrane is created by a phase inversion method as in the previous examples). The main disadvantage of this method is the same as the one previously mentioned regarding the use of spacers in hot pressing.

**Figure 8:**
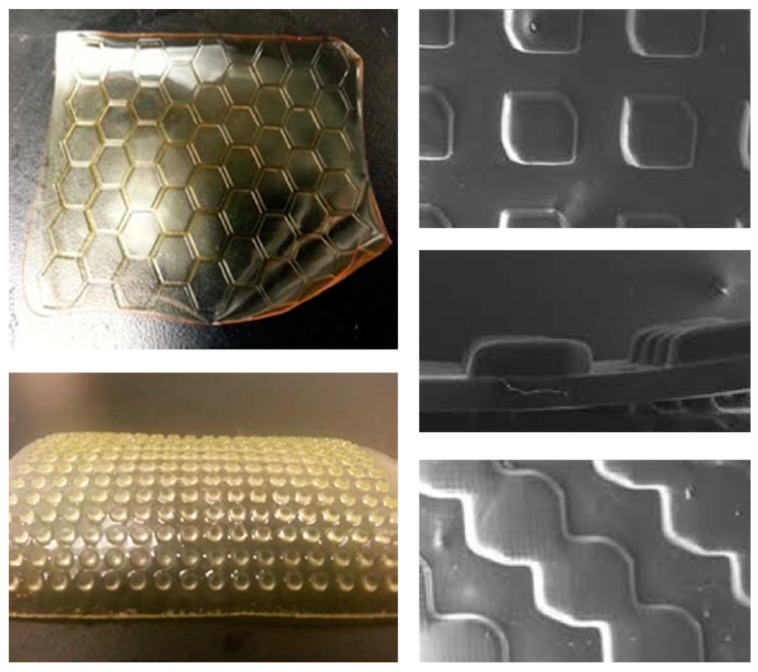
Illustrative examples (from References [52,60] of micro-patterned profiled ion exchange membranes prepared by 3D printing, (Reproduced with permission, Copyright 2016 and 2018, respectively, American Chemical Society).

Finally, 3D printing enables almost infinite possibilities for rapid prototyping of target materials, with different shapes and in different fields, by a number of available methods [57,58]. Considering fabrication of profiled ion exchange membranes, the most promising technique seems to be photopolymerization due to an easier and “greener” way of preparing ion exchange membranes by this method [59]. Seo et al. [52] prepared profiled AEMs via a photoinitiated free radical polymerization and quaternization process. A photocurable formulation (a mixture of, commercially available, oligomers, functional monomers and photoinitiators), can be directly cured into patterned films using a 3D photolithographic printer (Figure 7c). Depending of the initial composition of photocurable formulation, the final membrane material properties such as water uptake, permselectivity (which are all independent of a membrane being flat or profiled) and the ionic resistance (which is lower for profiled patterned membranes when compared with that of flat membranes of the same material volume) can be optimized [52]. Such an approach of preparing profiled ion exchange membranes is fast, solvent-free and occurs at ambient temperature. In any case, there is still a large margin for future progress in 3D printing of profiled membranes since, due to bleeding during curing, replication errors and a low-profile resolution are currently frequent [60]. Alternatively, the corrugations can be deposited on the top of a commercially-available membrane [53] (Figure 7d). 3D printing opens also an exciting new opportunity to create homogeneous profiled membranes with different shapes (Figure 8), which can be firstly designed and optimized by CFD simulations. 3D printers receive information about an object to be printed in a form of an .stl (stereolithography) file, which can be also used to define the geometry of computational domain in CFD studies [61]. Therefore, it becomes possible to change the information saved in .stl files and simulate fluid behaviour in channels formed by different corrugations, and then just print those profiled membranes, which CFD simulated performance would be promising.

## 6. Conclusions

Development of profiled ion exchange membranes have seen significant improvements over the last decade. So far, they have been already applied in research studies for electrodialysis, electrodeionization, reverse electrodialysis, and diffusion dialysis. In all these electromembrane-based processes, the utilization of profiled membranes improved their performance but, especially notorious, are their beneficial effects in electrodialysis. At high flow rates and current densities, profiled membranes facilitate formation of electroconvective vortexes, which enhance mass transfer and reduce deposition of foulants. Other mechanisms, which also promote ion transport enhancement are: lower water splitting rate; intensified hydrodynamic conditions, which lead to a decreased diffusion boundary layer thickness; and increase of active membrane area due to elimination of non-conductive spacers presence which also leads to a lower electric resistance. Thus, ultimately, profiled membranes should be designed with the objective of promoting convective phenomena. To achieve this, there is a large freedom in designing and testing (experimentally and/or by CFD simulations) novel forms, geometries, and shapes of corrugations. In a near future, manufacturing of such profiled membranes, with or without complex geometries, might see a significant progress due to the rapid advent of methods such as 3D printing. Novel applications of profiled ion exchange membranes are also envisaged in emerging processes, such as membrane capacitive deionization (MCDI).

## Abbreviations

AEM	Anion exchange membrane
CDI	Capacitive deionization
CEM	Cation exchange membrane
CFD	Computational fluid dynamics
ED	Electrodialysis
IEM	Ion exchange membrane
MCDI	Membrane capacitive deionization
OCF	Overlapped crossed filaments
PTV	Particle tracking velocimetry
Re	Reynolds number
RED	Reverse electrodialysis
SEM	Scanning electron microscope
